# An Experimental Study of the Effects of External Physiological Parameters on the Photoplethysmography Signals in the Context of Local Blood Pressure (Hydrostatic Pressure Changes)

**DOI:** 10.3390/s17030556

**Published:** 2017-03-10

**Authors:** Hongwei Yuan, Sven Poeggel, Thomas Newe, Elfed Lewis, Charusluk Viphavakit, Gabriel Leen

**Affiliations:** Optical Fibre Sensors Research Centre, Department of Electronic & Computer Engineering, University of Limerick, Limerick V94 T9PX, Ireland; Sven.Poeggel@ul.ie (S.P.); Thomas.Newe@ul.ie (T.N.); Elfed.Lewis@ul.ie (E.L.); Charusluk.Viphavakit@ul.ie (C.V.); Gabriel.Leen@ul.ie (G.L.)

**Keywords:** accelerometer, autonomic nervous system, local blood pressure, motion artefact, photoplethysmography (PPG), wearable biosensor

## Abstract

A comprehensive study of the effect of a wide range of controlled human subject motion on Photoplethysmographic signals is reported. The investigation includes testing of two separate groups of 5 and 18 subjects who were asked to undertake set exercises whilst simultaneously monitoring a wide range of physiological parameters including Breathing Rate, Heart Rate and Localised Blood Pressure using commercial clinical sensing systems. The unique finger mounted PPG probe equipped with miniature three axis accelerometers for undertaking this investigation was a purpose built in-house version which is designed to facilitate reproducible application to a wide range of human subjects and the study of motion. The subjects were required to undertake several motion based exercises including standing, sitting and lying down and transitions between these states. They were also required to undertake set arm movements including arm-swinging and wrist rotation. A comprehensive set of experimental results corresponding to all motion inducing exercises have been recorded and analysed including the baseline (BL) value (DC component) and the amplitude of the oscillation of the PPG. All physiological parameters were also recorded as a simultaneous time varying waveform. The effects of the motion and specifically the localised Blood Pressure (BP) have been studied and related to possible influences of the Autonomic Nervous System (ANS) and hemodynamic pressure variations. It is envisaged that a comprehensive study of the effect of motion and the localised pressure fluctuations will provide valuable information for the future minimisation of motion artefact effect on the PPG signals of this probe and allow the accurate assessment of total haemoglobin concentration which is the primary function of the probe.

## 1. Introduction

Photoplethysmography (PPG) is an optically recorded plethysmogram obtained from the human body by application of an external optical probe or measurement device. PPG sensors can measure spectral absorption of haemoglobin derivatives non-invasively and in real time [1,2,3,4,5,6]. PPG sensor devices normally consist of one or several remote sensor units for signal collection (e.g., finger sensor probe) and a central control unit for processing. Prior research by some of the authors of this investigation has demonstrated promising application of a custom designed PPG sensor device in monitoring heart rate (HR), oxygen saturation (SpO_2_) and total haemoglobin concentration (Hb) [3,4]. However, a drawback of this technology has been that the PPG signal is easily affected by motion artifact [5,6,7,8,9,10,11], requiring the subject (patient) to be motionless and lying or sitting down.

The movement of an individual wearing a PPG device can be classified into several different types. Yan et.al. proposed finger motion in five different positions, e.g., horizontal movement of finger and waving hand [5], and Peng et al. studied four types of finger motion, such as bending and pressing of the finger [6]. Methods used to minimize the impact of motion artefact include: adaptive noise cancellation algorithms (e.g., normalized least-mean-square) to recover signals corrupted due to body motion using acceleration data [7,8], relative sensor motion via self-mixing interferometry in a laser diode [9], periodic moving average filter [10], a method that uses synthetic noise reference signal without any extra hardware [11], etc.

The Autonomic Nervous System (ANS) of the human body, as part of the peripheral nervous system, can be disturbed by body motion events, leading to unconsciously varying vital physiological parameters including body temperature, blood pressure, respiration rate, heartbeat etc. [12]. The human ANS acts to achieve a balance between parasympathetic and sympathetic nervous systems. The sympathetic nervous system tends to adjust physiological parameters, such as increasing heart rate and blood pressure in a human body, in response to external/internal stimulating events; on the other side, the parasympathetic nervous system tends to release tension by reducing heart beat and blood pressure [13,14,15]. Research has been reported that the skin vasomotion is also modulated by the sympathetic nervous system [16]. The PPG signal, as a recordable bio signal, can also be affected by ANS when accompanied by variations in physiological events e.g., exercise, breathing and motion in general.

This paper reports on research relating to the effects of local BP variation [17], with a particular emphasis on the role of the ANS on the PPG signals. Section 2 describes the experimental setup and provides a schematic representation of the clinical PPG system used in these evaluations. The study of baseline and pulse amplitude from the viewpoint of local blood pressure variation is provided in Section 3. In Particular the corruption and/or degradation of the PPG signal is investigated when affected by local blood pressure variation induced from motion of the subject. In Section 4, other potential factors affecting the PPG signal are studied in detail e.g., Body Position, Breathing Pattern, Externally Applied Pressure, Motion at the wrist. Section 5 takes a further step for the measurements of PPG signals in the presence of more vigorous movement e.g., repeated swinging of the arm with long or short resting time intervals. The discussion and conclusions are presented in the last two sections.

## 2. Experimental Setup

The special purpose transmission-type PPG finger probe used in the experiments described in this article was recently designed and fabricated in order to study PPG signals in conjunction with motion. For this reason, an ADXL345 digital 3-axis MEMS accelerometer (ADXL345, Analog Devices, Cambridge, MA, USA) has been embedded within the PPG finger probe. It measures the static acceleration of gravity in tilt-sensing applications (for standard gravity, 1 g ≈ 9.81 m/s^2^), as well as dynamic acceleration resulting from motion and shock. The PPG finger probe can be divided into four parts: an LED, a photodiode, an acceleration unit and a processing circuit. The ADXL345 accelerometer can measure the motion in the PPG finger probe in three orthogonal directions (corresponding to *X*, *Y* and *Z* axes). The normal orientation of the PPG finger probe has been defined to be such that the positive side of the accelerometer reference *Z*-axis points in the upright direction (against gravity), the *X*-axis points along the finger (when held horizontal), and the *Y*-axis is orthogonal to the *X*-axis in the horizontal plane. Each experiment was conducted in the condition that the PPG finger probe was in the normal position and the subject had been sitting straight having been relaxed for at least 4 min prior to any testing.

**Figure 1:**
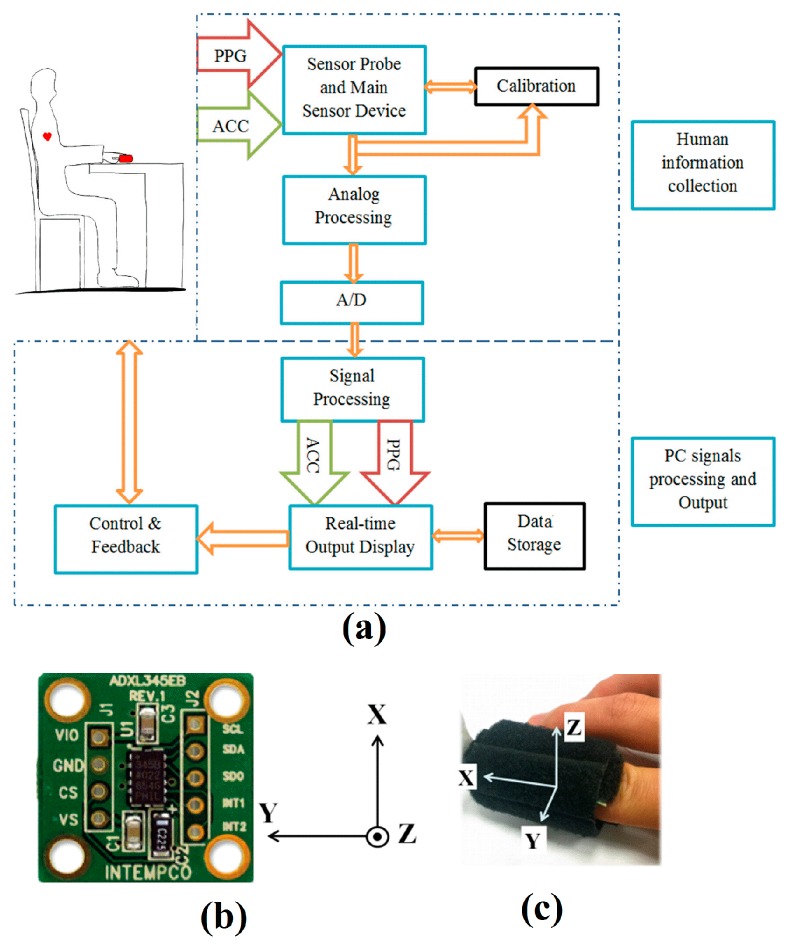
(**a**) Experimental setup of the custom sensor system; (**b**) Accelerometer and (**c**) Finger Clip with axes orientation.

The received optical signal at the photodiode is defined as the PPG signal and its intensity is represented by the PPG amplitude (Volts versus time (s). The detected signal at the output of the receiver amplifier (Volts) corresponds to the optical attenuation in that channel which comprises all losses including absorption, scattering and losses due to geometrical transmission. The finger sensor probe was attached to the subject using velcro tape in order to make the finger band size adjustable for different finger sizes. It is widely reported that the local blood pressure at the measuring point potentially introduces variability in measured PPG waveforms [18]. In all the following experiments, the PPG finger probe was placed on the right hand index finger in the same position in order to avoid the error from external pressure variations between the finger and the probe potentially caused by the need for over tightening or loosening the probe during attachment. The experimental data was initially recorded on five subjects with PPG signals, acceleration data and respiration monitored simultaneously. This was subsequently widened to a test on 18 independent subjects in order to confirm the efficacy of the initial results. Data has therefore been gathered for a total of 23 healthy adults (13 males and 10 females) between the age of 24 and 55 years (mean age 27.5 ± 7.5 years) with PPG signals, acceleration data and respiration monitored simultaneously. The room temperature was maintained constant around 18 °C, and all subjects had no known cardiovascular diseases, nor recent consumption of caffeine and/or nicotine. The schematic showing a block diagram representation for the experimental setup of the custom sensor system is illustrated in Figure 1a and the finger clip sensor illustrating the axes orientation in Figure 1b,c.

PPG and acceleration (ACC) signals are collected from the sensor device and later analyzed for human vitals monitoring. LabVIEW™, a digital signal processing software package (National Instruments, Austin, TX, USA), was used to compute the various optical absorption parameters, accelerometer coefficients and the derived coefficients (e.g., (SpO_2_), Hb and HR). A study of the effects of motion of the probe on the PPG signal shows that the PPG signal is potentially corrupted/degraded by movement, and strongly affected by height variation in vertical direction. A blood pressure monitor (SBC 27-wrist blood pressure monitor, Sanitas, Kaunas, Lithuania) was used to simultaneously measure the local BP in close proximity to the point of measurement when the subject’s hand was placed at different height levels. A respiration monitor, (Neulog Respiration Monitor Belt logger NUL-236, Fisher Scientific, Pittsburgh, PA, USA) was also included for monitoring the subject’s breathing in real time during testing. It is a piezoresistive device used to measure the deformation in a bellows diaphragm when an internal air cavity in the belt is compressed by stress due to abdominal expansion when breathing.

All subjects gave their informed consent for inclusion before they participated in the study. The study was conducted in accordance with the Declaration of Helsinki, and the protocol was approved by the Ethics Committee of 2016_11_04_S&E.

## 3. Baseline and Local BP

### 3.1. Baseline of PPG Signal Strongly Affected by Acceleration in the Vertical Direction

**Figure 2:**
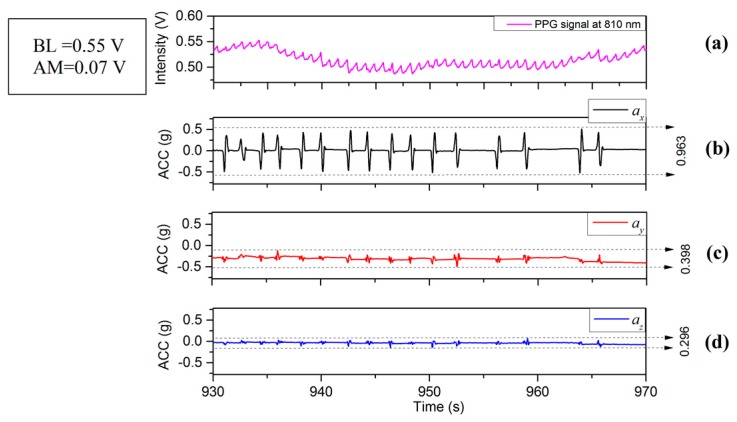
(**a**) PPG and (**b**–**d**) ACC signals with the main motion acceleration in *X*-axis.

**Figure 3:**
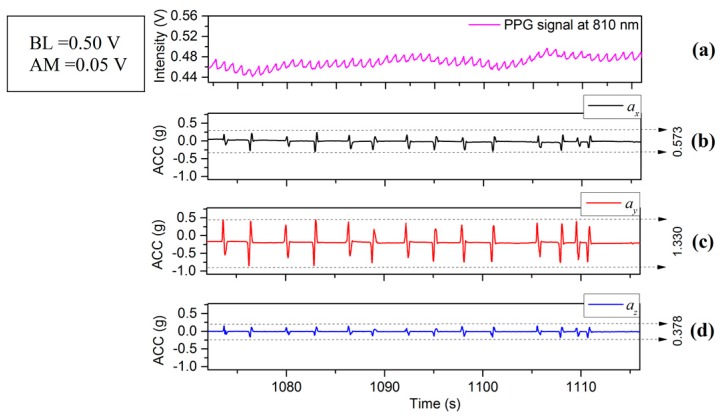
(**a**) PPG and (**b**–**d**) ACC signals with the main motion acceleration in *Y*-axis.

**Figure 4:**
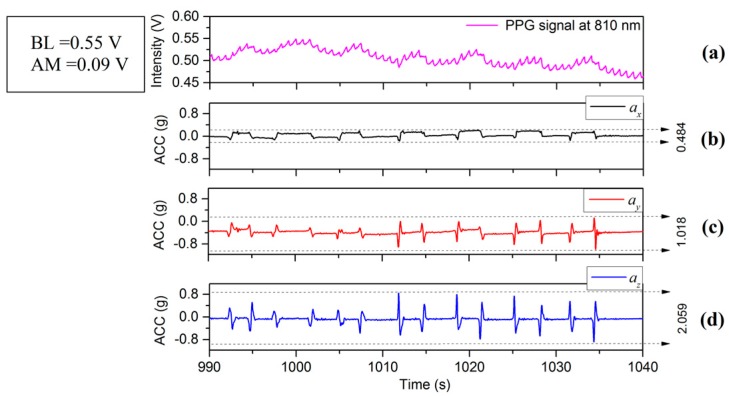
(**a**) PPG and (**b**–**d**) ACC signals with the main motion acceleration in *Z*-axis.

**Figure 5:**
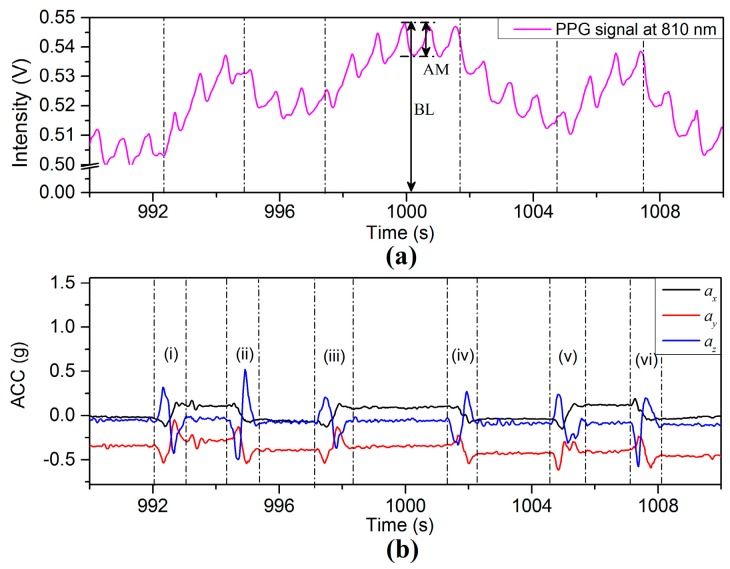
Enlarged portion of Figure 4 in the interval 1290–1310 s: (**a**) PPG and (**b**) ACC signals.

Figure 2, Figure 3 and Figure 4 depict the time varying PPG signals at a wavelength of 810 nm and ACC signal the accelerometer signals (ACC) signal measurements with predominant acceleration applied in the *X*, *Y*, and *Z* directions respectively. The results of Figure 2, Figure 3 and Figure 4 show that the design of the PPG probe in this investigation has been successful and there appears to be minimal effect accruing from motion on the PPG signals in the respective directions (*X*, *Y* and *Z*). Figure 2 corresponds to the case of the motion applied in the *X*-axis direction, in which case accelerations applied in the *Y*-axis and *Z*-axis directions were controlled to be as small as possible. The resulting acceleration signals are shown in Figure 2a (*X*) and Figure 2c,d (*Y* and *Z* respectively). The acceleration in the *X* direction, $a_{x}$ is $0.963\;g\;\left(\approx 9.45\;m/s^{2}\right)$ whereas $a_{y}$ (*Y* direction) and $a_{z}$ (*Z* direction) are $0.398\;g\;\left(\approx 3.90\;m/s^{2}\right)$ and $0.296\;g\;\left(\approx 2.90\;m/s^{2}\right)$. The time varying PPG signal during this motion event is shown in Figure 2a and the variation of mean values is affected by a lower frequency component mainly from respiration [19]. Nitzan et al. [20] have also stated “Photoplethysmography (PPG) provides a qualitative measure of the tissue blood volume increase during systole by measuring the light transmission through the tissue as a function of time.” The time period relating to our study is not fixed (Figure 2, Figure 3 and Figure 4) but is generally in the range of multiples of 10’s of seconds (usually between 20 and 30 s). In order to introduce a quantitative measure of the PPG signal change and (amplitude) and offset from the zero line (analogous to DC component) during this phase the definition has been applied to each record for the duration that is captured. The peak value in the PPG waveform, defined in terms of baseline (BL) (being the difference between 0 and the maximum value of this waveform during the interval) [20], and amplitude (AM) of a PPG signal, are also illustrated in Figure 5. Results of acceleration applied mainly along the *Y*-axis direction are illustrated in Figure 3. The acceleration $a_{y}$ is $1.330\;g\;\left(\approx 13.05\;m/s^{2}\right)$ whereas $a_{x}$ and $a_{z}$ are $0.573\;g\;\left(\approx 5.62\;m/s^{2}\right)$ and $0.378\;g\;\left(\approx 3.71\;m/s^{2}\right)$. However, when acceleration in the vertical (*Z*) direction is dominant, i.e., in Figure 4d with the acceleration $a_{z}$ is $2.059\;g\;\left(\approx 20.20\;m/s^{2}\right)$ whereas $a_{x}$ and $a_{y}$ are $0.484\;g\;\left(\approx 4.75\;m/s^{2}\right)$ and $1.018\;g\;\left(\approx 9.99\;m/s^{2}\right)$ respectively. The PPG signal (Figure 4a) therefore shows stronger fluctuations than the case in Figure 2a or Figure 3a. It therefore appears that PPG signals are more greatly influenced by acceleration applied in the *Z*-axis direction. It is acknowledged that the acceleration values in the case of Figure 4a are greater than either Figure 2a or Figure 3a, but the effect on the waveform of the zaxis acceleration is visibly different in the case of Figure 4a than either Figure 2a or Figure 3a, the signal in the former case showing a stronger oscillatory characteristic which broadly correspond the appearance of acceleration pulses and are not as clear or even present in Figure 2a and Figure 3a for which the dominant applied acceleration is in the horizontal plane.

The PPG waveforms and ACC signals in the interval 990–1110 s in Figure 4 are shown in greater detail in Figure 5. Each acceleration event in the positive *Z*-axis direction, marked by regions of odd numerals (i, iii, and v) in Figure 5b, leads to an increase in BL, and meanwhile each acceleration in negative *Z*-axis direction, marked by regions of even numerals (ii, iv, and vi), represents a decrease in BL. The waveform is therefore most strongly influenced by movement in vertical *Z*-axis direction compared to motion in the horizontal x-y plane. The research in this investigation is aimed at the exploration and analysis of the properties of the PPG signal affected by physiological parameters associated with motion as described in the following sections.

### 3.2. BP Near the Finger Probe Response to Arm Position

Inflatable cuff blood pressure monitors are widely available for BP and HR monitoring. An inflatable cuff blood pressure monitor performs well when the monitored subject is in the resting position. The average values of systolic BP, diastolic BP and HR from one of the subjects were collected at different height levels (i.e., the height of the wrist, that the measurement cuff is on, relative to heart level) using the Sanitas SBC 27-wrist blood pressure monitor. The results corresponding to each of these parameters with differing height level are shown in Table 1. The instructions provided for the blood pressure monitor direct that measurements should be recorded with the cuff located at the same height as heart level in order to obtain optimum accurate readings, which means the first set of readings in Table 1 should be the most accurate. Although increasing vertical height difference (VHD, the vertical distance from the attached PPG probe to heart level) is not a recommended way to use the blood pressure monitor, these readings were recorded to provide an indication of variation in local BP variations. The results of Table 1 clearly show that an increase of VHD results in lower BP measurements. In detail, the systolic BP decreases about 5 mmHg for every 5 cm increase in height above heart level, fall from 112 mmHg to 89 mmHg over the entire recording interval (35 cm). The diastolic BP readings show a similar decrease from 84 mmHg to 45 mmHg over the same height range. In the situation where VHD increases, HR readings remain relatively stable (a second reference for the HR reading was also available from the Oxygen Saturation Monitor PULSOX-8, Minolta, Osaka, Japan). These results demonstrate that change of VHD affects the local BP due to blood volume change at the measuring site. It is well known [21,22] that a variation in BP of 2 mmHg for every 25 mm can occur above or below the heart level and therefore for every 50 mm it can be extrapolated that a BP variation of 4 mmHg would occur, which is in good agreement with the results of this investigation. This variation is well known and established, but provides efficacy of the results recorded for these parameters in this investigation.

## 4. Factors Which Effect the PPG Signal

The PPG signal is a qualitative measure related to blood volume changes at the point of measurement site, the fingertip being the most widely adopted measurement site due to the wide distributed peripheral blood circulation. It is clear from Figure 2a, Figure 3a and Figure 4a that the PPG signal includes a higher frequency component (circa 1–1.5 Hz under normal light physiological stress conditions) which synchronizes with the cardiac system attributed to each heartbeat as well as various other lower frequency components e.g., variations due to respiration. The PPG signal is easily affected by various factors resembling that of affecting the blood volume in the measurement area [16]. These factors could be summarized as variation of local BP induced from the change of blood flow in the measurement site and are described below.

### 4.1. Vertical Height Difference (VHD) Variation

**Figure 6:**
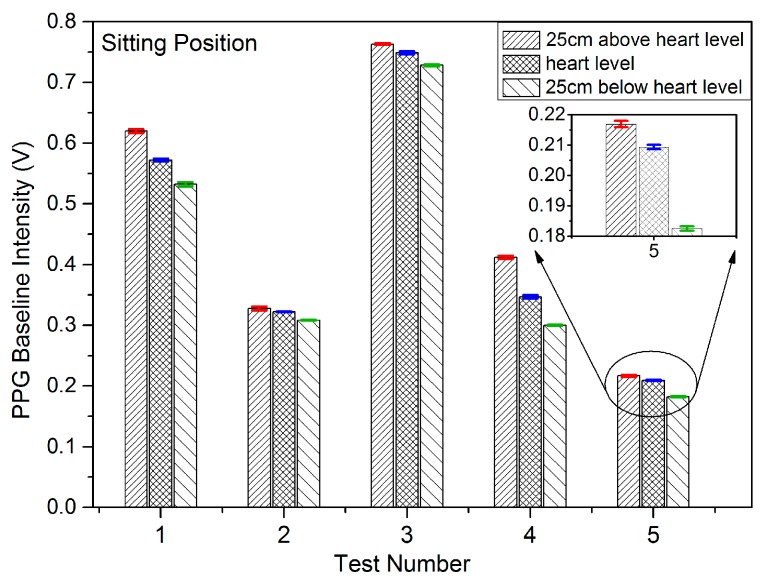
PPG baseline intensities and standard deviations when the subjects were in the sitting position and the finger sensor probe set at three different vertical levels.

**Figure 7:**
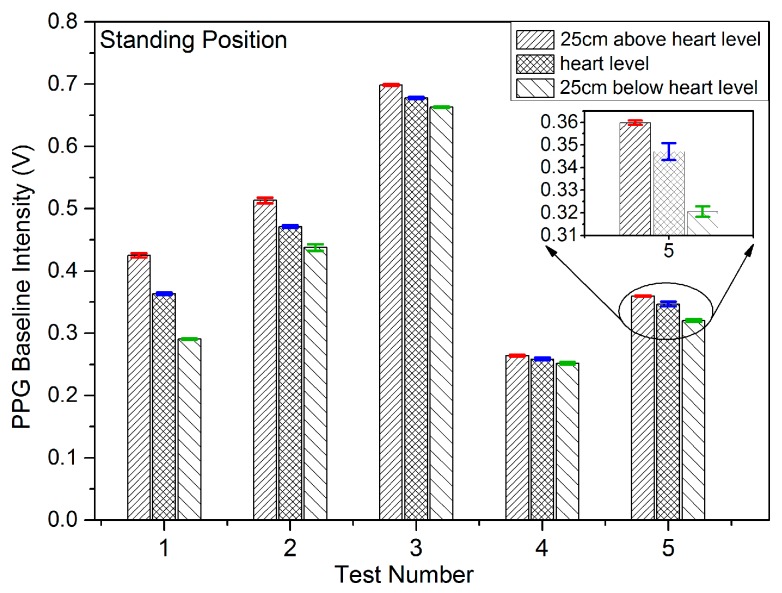
PPG baseline amplitudes and standard deviations when the subjects were in the standing position and the finger sensor probe set at three different vertical levels.

**Figure 8:**
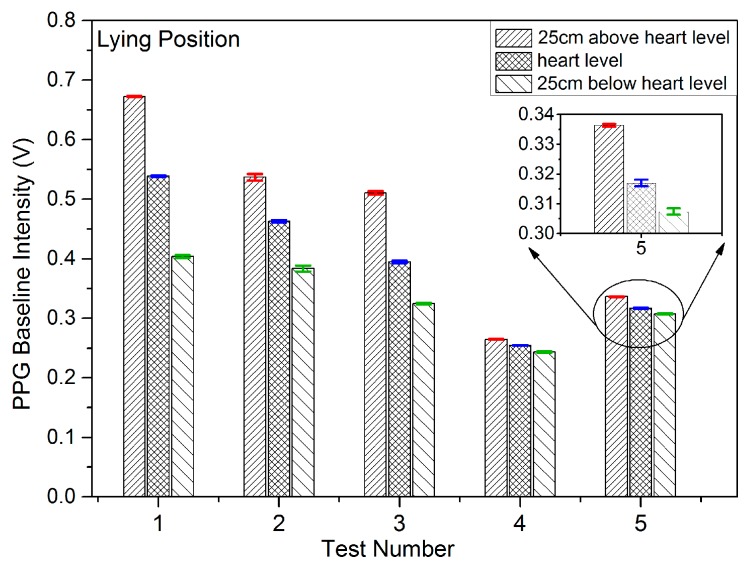
PPG baseline amplitudes and standard deviations when the subjects were in the lying position and the finger sensor probe set at three different vertical levels.

Results were recorded initially for five subjects being in the sitting, standing and lying positions in order to study the effect on the PPG signals of VHD variation. In each measuring position, the height of the finger sensor probe was set at three different levels, 25 cm above heart level, at heart level, and at 25 cm below heart level with support for 2 min in each position. The PPG sensor probe was maintained in the normal position on the finger at all times. The PPG signals from the five subjects were captured at the three different vertical levels for which the subjects were in the sitting position, standing position, and lying position. Figure 6, Figure 7 and Figure 8 illustrate the data of these tests for each case of body position respectively.

Further set of measurements were recorded for 18 additional subjects which were healthy adults (9 Males and 9 females). Data were recorded for each of the body positions referred to above, but due to limitations of available space the data set is represented for only the sitting position. The results from each of these tests are captured in Table 2.

The studies of PPG baseline amplitudes are shown as histograms with standard deviations in Figure 6, Figure 7 and Figure 8, for which the subjects were in sitting, standing and lying positions, respectively. The Mean BL and AM values in Table 2 are the mean of the maximum and minimum BM and AM values across a single record and the ±SD is the standard deviation in that value over the entire duration of the record (typically in the range 20 to 30 s) around the mean level. The standard deviation in this case represents the variation of the signal in a single time frame as illustrated in Figure 5a. The results of Figure 6, Figure 7 and Figure 8 clearly demonstrate a consistent decrease of BL in the PPG signal with the decrease of positive VHD deviation. These observations demonstrate that the PPG signals reflect blood volume changes (hydrostatic pressure changes) in tissue when the BP near the finger probe (at the wrist) is varied with respect to VHD. The PPG signals at the point of measurement are clearly affected by the VHD between the finger probe and heart level.

**Figure 9:**
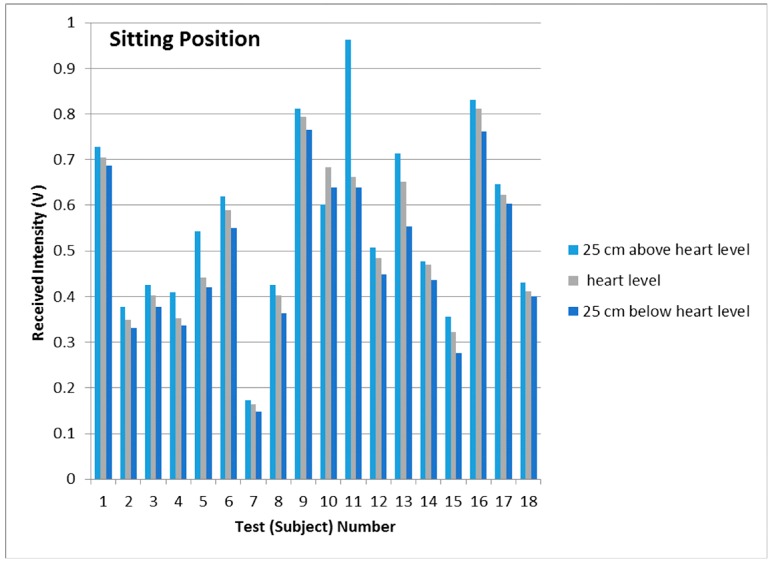
PPG baseline amplitudes for 18 subjects whilst in the sitting position and the finger sensor probe set at three different vertical levels.

The data sets, mean and median of BLs vs. VHD deviation are represented graphically in Figure 9. It is clear that the BLs of the PPG signals increase with increasing positive VHD deviation from 0 cm at heart level to 25 cm above heart level and likewise decrease with increasing negative VHD deviation from 0 cm at heart level to 25 cm below heart level. This is true for all 18 cases measured with the exception of subject number 10 which includes a single anomalous result. Taken in conjunction with the results of Figure 6, this means that results from 22 out of 23 subjects have exhibited this consistent trend. It can therefore be deduced that the BP near the finger sensor probe consistently decreases with increasing positive VHD deviation and increases with the increasing negative VHD deviation. Furthermore, in the case of increasing VHD deviation, the BP at the point of measurement tends to decrease (Table 1) whilst the BL of the PPG signal tends to increase (Figure 6 and Figure 9); conversely for increasing negative VHD deviation, the BP near the finger sensor probe tends to increase while BL of PPG signal tends to decrease.

A preliminary statistical analysis involving calculation of the mean, median, the standard deviation and interquartile range of BL and AM from the 18 sets of PPG signals at Different VHD Deviation was undertaken on the results of Table 2. These are presented in summary form for BL and AM of the PPG signal in Table 3 and Table 4.

### 4.2. Autonomic Nervous System (ANS) Variation Induced from Physiological Events

Dynamic change of blood flow and arterial BP during postural change from sitting to standing has been discussed by Olufsen, et al. [23]. ANS variation induced from physiological events tends to vary the blood flow and BP, as well as the local BP near the sensor probe.

#### 4.2.1. Changing Body Position

**Figure 10:**
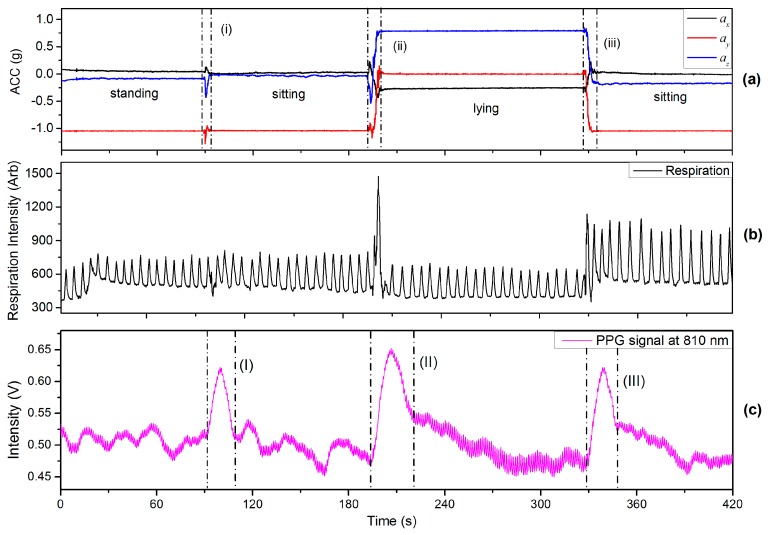
(**a**) ACC; (**b**) respiration and (**c**) PPG signals recorded from changing body positions.

This section includes the results of an experiment in which the finger sensor probe was fixed at the same VHD whilst changing the body position. In one of the experiments the finger sensor probe was kept in front of chest at heart level whilst changing body positions between sitting, standing and lying positions. The subjects were requested to breathe evenly and smoothly without talking throughout the experiments. The recorded respiration data corresponding to the arbitrary analog output of the respiration monitor belt device is shown in Figure 10b. The period from the rising edge of PPG signal to the start of a stable region (no significant signal change) is defined as a PPG-fluctuation, e.g., the regions (I–III) in Figure 10c. Region (i) in Figure 10a indicates a sitting down process (from standing to sitting). The reason for the PPG-fluctuation in Figure 10c is believed to arise from ANS variations induced by the change in body position. Since there is no VHD, the observed values of BLs are almost the same before and after the PPG-fluctuation as was to be expected. Region (ii) represents the process of lying down for which the height of the finger sensor probe was unavoidably changed from heart level to a level about 6 cm above heart level since the finger sensor probe was kept in front of chest at all the times. The PPG fluctuation [region (II in Figure 10c)] is believed to be caused by an ANS variation accompanied with BP changes resulting from hydrostatic changes caused by the body position variation. Similarly, region (iii) corresponds to the postural change from lying to sitting, and another PPG-fluctuation [region (III)] is associated with it. Rotating the wrist was also included in this experiment, indicated by region (ii) and (iii), in Figure 10a. The effect of wrist rotation on PPG signals is discussed in detail in Section 4.4 of this paper.

Results were initially gathered from five separate randomly selected subjects for which body position varied from a standing position to a sitting position corresponding to the tests outlined above. These are characterised using the parameters outlined in Table 5. These parameters are defined as the period of body variation process as *t*_∆ACC_ (in seconds); the interval of the PPG fluctuation according to body position variation as *t*_∆BL_ (in seconds); respiration rate (RR) (in units of per min); and the difference between the peak value of BL in the fluctuation and average of BL before the fluctuation (a 60 s interval used in this case) as ∆BL (in Volts). The demonstration shows that the PPG signals at the point of measurement are affected by the ANS variations induced by change in body position.

Further independent tests results were obtained from the other 18 subjects referred to in Section 4.1. For which body position was varied from a sitting position to a standing position and these are listed in Table 6, accompanied with a study of the preliminary statistical analysis including mean, median, standard deviation and interquartile range as shown in Table 7. These results captured for a large group of subjects confirm the efficacy of the earlier results (Table 5) and hence the validity of the measurement technique employed in this investigation.

The experimental results of this section show the PPG fluctuation as a result of body positional changes. A position change involves a change in blood flow and BP, and homeostasis is recovered by means of the ANS. The PPG fluctuation is therefore most likely caused by hemodynamic changes and not solely by the ANS. Furthermore it is generally accepted that the parasympathetic system dominates whilst in the lying position, and therefore the effect of the ANS should be present throughout the duration of the lying position.

**Figure 11:**
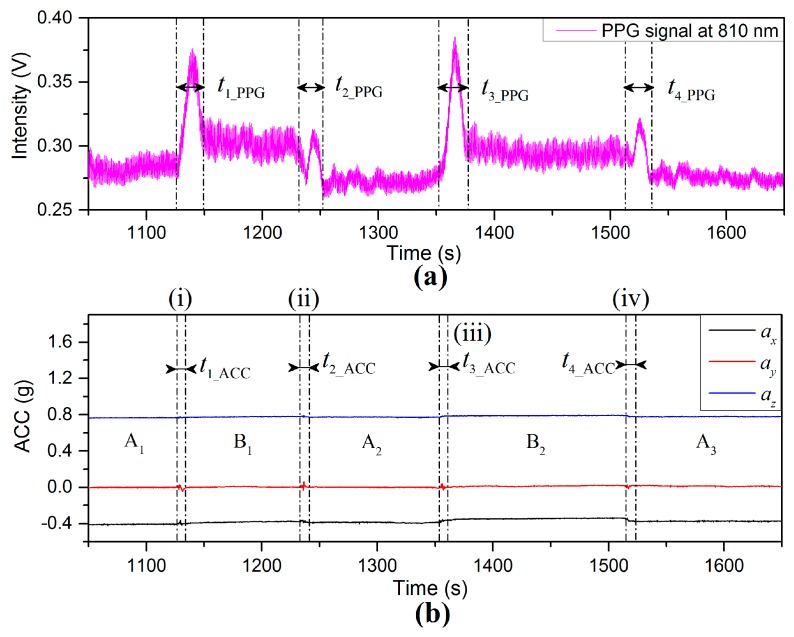
(**a**) PPG and (**b**) ACC signals for which the subject maintained the finger sensor probe at the same height above the floor and varied the VHD by changing body positions.

PPG and ACC time dependent signals are shown in Figure 11 for which the subjects were required to maintain the finger sensor probe at the same height above the floor and the VHD was varied by changing body position. Time period $A_{1}$ represents the subject standing still with the finger sensor probe at 25 cm below heart level. Region (i) represented by $t_{1_{\mathrm{ACC}}}=7.6\;s$ in Figure 11b shows the process of sitting down slowly and the VHD changing from 25 cm below heart level to a level close to heart level, where the amplitudes of accelerations are low and in the range of 0.05 g (0.49 ms^−2^). Referring to Figure 11a, it can be seen that there is an increase of BL in the PPG signal during the time period defined by $t_{1\_\mathrm{PPG}}=22.7\;s$. The time duration difference between $t_{1\_\mathrm{ACC}}$ and $t_{1\_\mathrm{PPG}}$ is 15.1 s, however during $t_{1\_PPG}$ the BL fluctuates significantly whereas the accelerometer on the finger sensor probe detects only limited acceleration due to motion. Time period $B_{1}$ corresponds to signals recorded for which the subject was sitting with the finger sensor probe at heart level. Region (ii) which is defined by $t_{2\_\mathrm{ACC}}=6.7\;s$ in Figure 11b shows the accelerometer signal while the subject was standing up and the VHD changes from heart level to 25 cm below heart level. During the interval (ii), the BL of the PPG signal decreases, and the time interval for the PPG signal change is described by $t_{2\_\mathrm{PPG}}=20.3\;s$. There is a 13.6 s difference between $t_{2\_\mathrm{ACC}}$ and $t_{2\_\mathrm{PPG}}$. The transition intervals (iii) and (iv) thereafter display similar results to those of (i) and (ii), being 18.8 s and 18.0 s difference, respectively, between the accelerometer data settling and the PPG signal settling.

**Figure 12:**
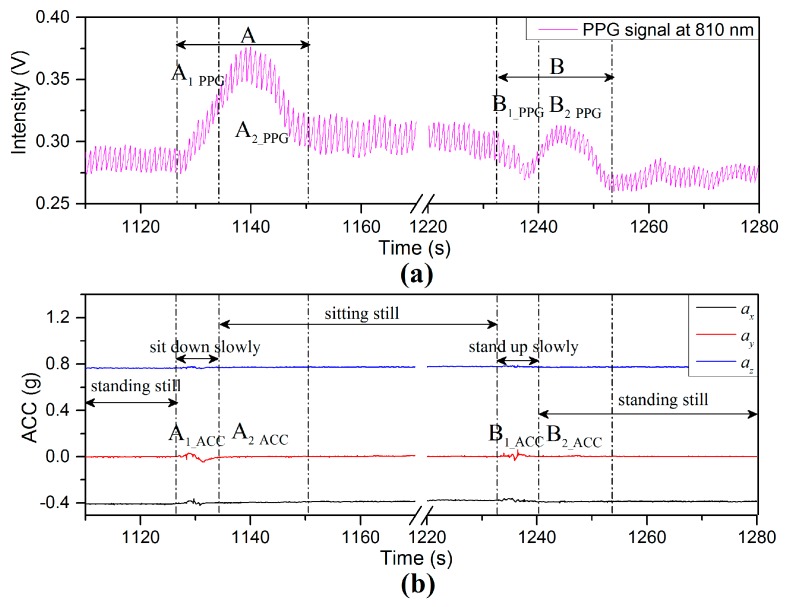
Enlarged portion of the PPG (**a**) and the ACC signals (**b**) in the interval 1110–1280 s in Figure 11.

The details of the PPG and the ACC signals in the interval 1110–1280 s in Figure 11 are represented in Figure 12. The PPG fluctuation in Region (A) is the period from the rising edge of PPG signal to the start of a stable region. This interval can be divided into two regions for both signals: $A_{1\_\mathrm{ACC}}$ and $A_{2\_\mathrm{ACC}}$ for the ACC signals (Figure 12b; and these are transposed to $A_{1\_\mathrm{PPG}}$ and $A_{2\_\mathrm{PPG}}$ for the PPG signal in the case of Figure 12a. Region ($A_{1\_\mathrm{ACC}}$) defines the total interval during which the body position was changed from the standing to the sitting position. The decrease of VHD between the finger sensor probe and heart level is 25 cm due only to change of body position (while the finger sensor probe was kept still and stayed at the same height above the floor). The BP near the finger sensor probe decreases with decreasing negative VHD deviation according to the previous result in Section 3.2 and the increase of BL according to the previous result in Figure 9, as shown by the region $A_{1\_\mathrm{PPG}}$ in Figure 12a. Region $A_{2\_\mathrm{ACC}}$ (Figure 12b, is the interval where the accelerometer on the finger sensor probe detects only limited or no acceleration due to motion, while the BL of component $A_{2\_\mathrm{PPG}}$ initially increases (dominated by the sympathetic part of the ANS) and then decreases (dominated by the parasympathetic part) during which time the homeostasis process is recovered via the ANS [20,24].

Region (B) in Figure 12 is the interval from the decreasing edge of the PPG signal to where it stabilizes again. This interval can be divided into two regions for both signals: $B_{1\_\mathrm{ACC}}$ and $B_{2\_\mathrm{ACC}}$ for the ACC signals; $B_{1\_\mathrm{PPG}}$ and $B_{2\_\mathrm{PPG}}$ for the PPG signal. Region ($B_{1\_\mathrm{ACC}}$) is the interval during which the body position was changed from sitting to standing position. The increase of VHD is 25 cm (while the finger sensor probe was kept still and stayed at the same height above the floor). The BP falls with the decrease of VHD (consistent with the data of Figure 9), leading to the decrease of BL, as shown by region ($B_{1\_\mathrm{PPG}}$). Region ($B_{2\_\mathrm{ACC}}$) defines the interval where the accelerometer on the finger sensor probe detects limited or no acceleration due to motion, while the BL of component $B_{2\_\mathrm{PPG}}$ firstly increases (dominated by sympathetic part of the ANS) and then decreases (dominated by parasympathetic part) during which time the homeostasis process is recovered via the ANS. The PPG fluctuation observed in region (B) is dominant and is induced by body position variations (governed by BP changes from hydrostatic changes in the body position variation) compared with increase of negative VHD deviation accompanied with blood volume changes and hydrostatic changes (increase of BP near the finger sensor probe induced by the VHD variation). Clearly, BL variations of components $A_{2\_\mathrm{PPG}}$ and $B_{2\_\mathrm{PPG}}$ are not affected by motion but by the increase of the BP at the point of measurement. It is conjectured that changing the body position leads to a physiological variation with hydrostatic pressure changes, resulting in an increase of BP, which stimulates a response of the ANS [2,20].

#### 4.2.2. Changes in the Breathing Pattern

PPG signals are sensitive to physiological variations; several examples are given in the literature [2], such as changes in the breathing pattern, coughing or yawning. Similar experiments were conducted for the current investigation and detailed results include the PPG waveforms resulting from these tests.

**Figure 13:**
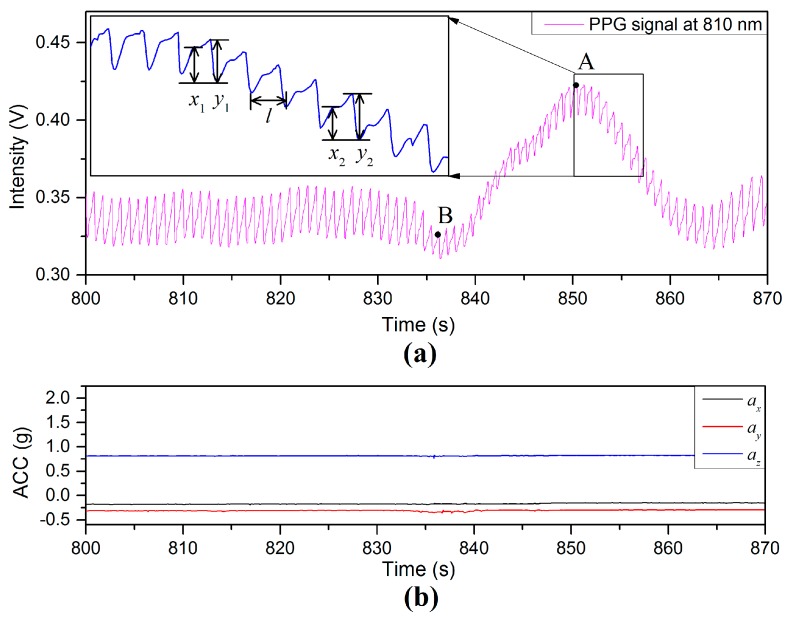
(**a**) PPG signal and (**b**) ACC signals with a deep yawn event.

Many types of physiological influences can result in an increase, or decrease of the local BP at the measuring site, which affects the BL and the AM of the PPG signals. Figure 13 illustrates PPG and acceleration signals during a deep yawn event. As can been seen from the intensity of PPG signal in Figure 13a, the average value of BL is 0.35 V before the deep yawn. The activity of a deep yawn started at the time of 835 s. The next 25 s shows a PPG-fluctuation triggered by the yawn event. Repeated experiments (40 times) show that: (1) the value of AM tends to be restricted and (2) the BL tends to continue without significant variation. A zoomed PPG signal in the time period of 852–857 s is included as an inset on the top left of Figure 13a. The interval l is the instantaneous period of the PPG waveform, at a given point in time, and has been used to determine HR. Two examples of the AM of the diastolic peak are illustrated by $x_{1}$ and $x_{2}$, whereas $y_{1}$ and $y_{2}$ are examples of the AM of the systolic peaks [25]. The mean ratio of diastolic peak to systolic peak during the period 810–830 s is 0.403 (±0.023) in Figure 13 prior to the yawning event. During the yawning event, the value of the ratio $x_{1}/y_{1}$ (e.g., 0.83 at the peak point, A) is larger than that of $x_{2}/y_{2}$ (e.g., 0.52 at the valley point, B), and the BL/AM ratio is also affected during the PPG fluctuation. Even though the PPG signal is highly affected by the yawn activity, the interval l can still be reliably used to calculate HR [26,27].

**Figure 14:**
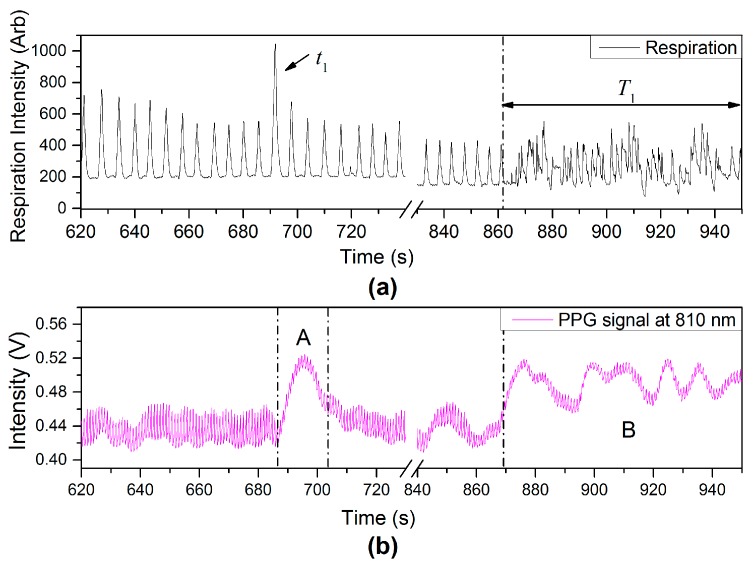
(**a**) Respiration including deep breathing and talking events and (**b**) PPG signal.

Another type of physiological variation from variation of breathing pattern, deep breathing ($t_{1}$) and talking ($T_{1}$) events, can also affect the PPG signals as observed in PPG-fluctuations [region (A) and (B)], shown in Figure 14.

### 4.3. External Pressure at the Measuring Site

**Figure 15:**
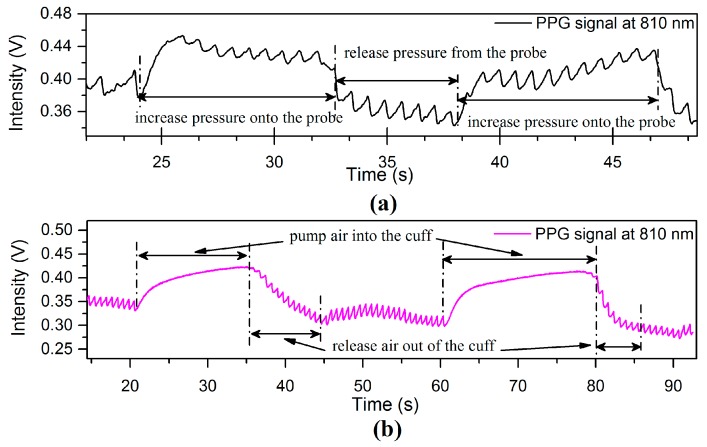
The PPG signal affected by local BP variations at the measuring site due to (**a**) external pressure applied on finger probe and (**b**) external pressure applied on wrist by processing the inflatable-cuff type blood pressure monitor.

Figure 15 shows other types of PPG-fluctuation caused by local BP variations at the point of measurement due to Figure 15a external pressure applied to the finger sensor probe and Figure 15b external pressure applied to the wrist by inflating a cuff type blood pressure monitor. According to the manufacturer’s/suppliers data sheet the blood pressure monitor was capable of applying continuous pressure on the wrist in the range of around 95–195 mmHg. The applied pressure from the cuff inflation tends to block perfusion, affecting the blood volume at the point of measurement, and after the release of pressure or cuff deflation, the amplitude of the PPG signal gradually returns to the pre-inflation level [16]. External pressure is therefore a third factor that can affect the PPG signal in addition to VHD and ANS.

### 4.4. PPG Signal Response to Wrist Rotation

**Figure 16:**
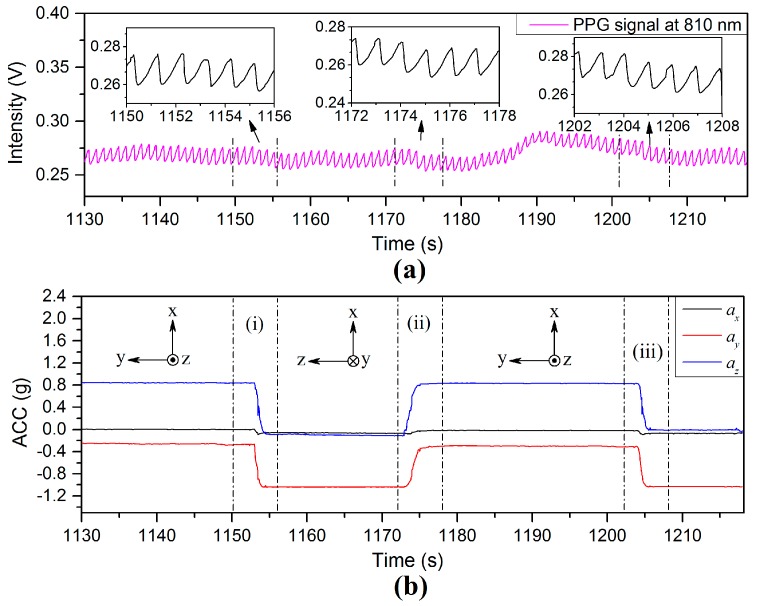
Illustration of (**a**) PPG and (**b**) ACC signals recorded from rotating wrist.

More experiments were performed to study the response of PPG signal to wrist rotation. The sensor probe on the finger was initially placed in the at heart level. One experiment was conducted by slowly rotating the wrist through 90° anticlockwise or 90° clockwise rotation along the *X*-axis (the direction which the finger is pointing), as shown in Figure 16. Region (i) shows the PPG signal for a 90° anticlockwise rotation of the wrist. The enlarged PPG waveforms (left) in the interval 1150–1156 s are slightly disturbed by motion artifact, but the values of BLs and AMs are relatively stable compared with those without the wrist rotation regions. Region (ii) shows a 90° clockwise rotation of the wrist. The enlarged PPG waveforms (middle) in the interval 1172–1178 s are minimally affected by the rotation process. Region (iii) is a repeat process of region (i), which has its detail shown in top right in Figure 16a. This experiment indicates that wrist rotation does not significantly affect the PPG signals.

## 5. Measurements When Swinging the Arm

Two experiments were conducted where the finger sensor probe was placed on the finger in line with the upper arm and forearm, and the subject’s arm was fully outstretched and held straight. 

### 5.1. Swinging Arm with Long Resting Time Intervals

**Figure 17:**
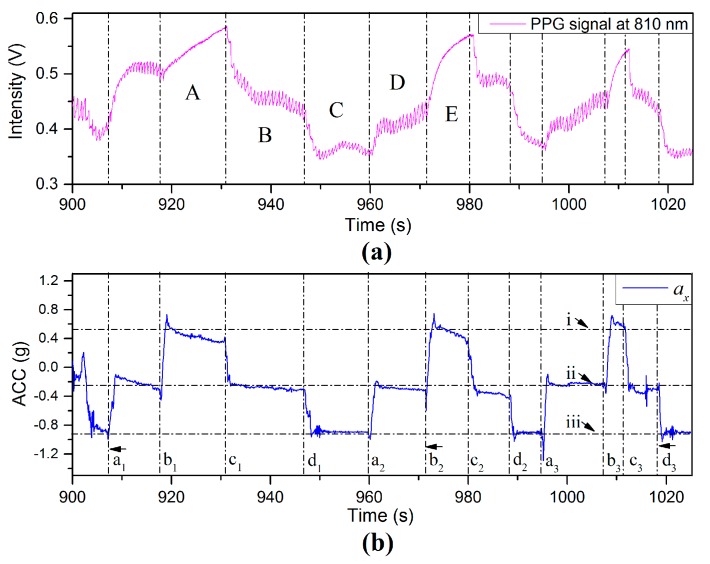
(**a**) PPG and (**b**) *X*-axis ACC signals captured in the process of swinging the arm up and down with long resting time intervals.

Figure 17a,b show the PPG and *X*-axis acceleration signals in the process of swinging the arm up and down by rotation at the shoulder. The positions of the finger sensor probe are indicated by the *a_x_* signals in Figure 17b, where dotted lines (i), (ii), and (iii) mark the sensor probe at 35 cm above heart level, heart level, and 35 cm below heart level, respectively. Dotted lines ($a_{i}$) mark the probe rising from 35 cm below heart level to the heart level, dotted lines ($b_{i}$) mark the start of the probe moving from heart level to 35 cm above heart level, dotted lines ($c_{i}$) mark the probe being lowered from 35 cm above heart level to heart level, dotted lines ($d_{i}$) mark the changes of the probe from heart level to 35 cm below heart, et al.

In the process of swinging the finger sensor probe up and down with long resting intervals (approximately 10 s), the values of the PPG intensity, in Figure 17a, vary in a manner similar to *a_x_* in Figure 17b. Region (A) is the stationary stage following lifting of the finger sensor probe to 35 cm above heart level. The BL of PPG signal shows a sharp increase and the AM is seriously degraded. The increase of positive VHD deviation results in significant attenuation of the pulsatile component. Region (B) shows the PPG signal with the finger sensor probe returning back down to heart level. The amplitude signal returns to its normal good quality after a short delay. When the finger sensor probe was set at 35 cm below heart level, indicated by Region (C), the AM of the PPG signal tends to be less than that at heart level. Region (D) delimits a period when the finger sensor probe was raised to heart level and the BL of the PPG increases with the increase of the *a_x_* value. Region (E) represents a repeat of the movements in region (A) but with a higher acceleration. An observation from this experiment is that the VHD between the finger sensor probe and heart level can be inferred from the *X*-axis acceleration. The PPG signals show large time delay before they reach a constant value during all the movements.

### 5.2. Swinging Arm up and down with Short Resting Time Interval

**Figure 18:**
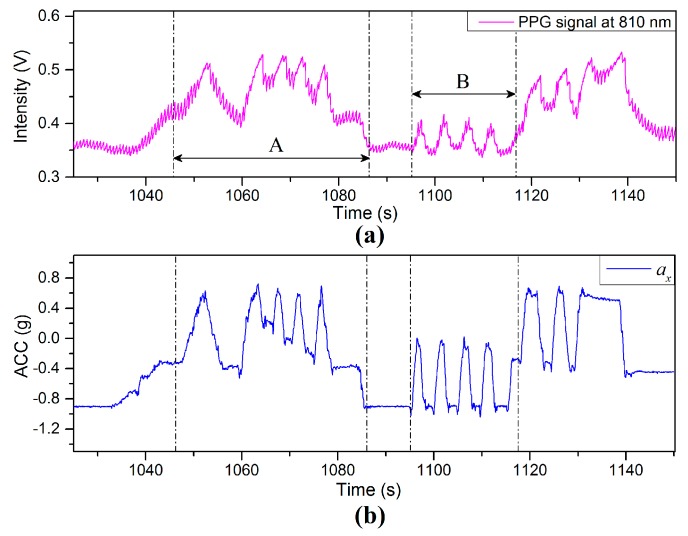
(**a**) PPG and (**b**) ACC signals captured in the process of swinging the arm up and down with short resting time intervals.

Figure 18 shows the PPG and acceleration signals captured in the process of swinging the arm up and down repeatedly with short resting time intervals (less than 5 s). In Region (A) the finger sensor probe is subject to rapid up-and-down motions between a height of 35 cm above heart level and heart level. Region (B) corresponds to the finger sensor probe being quickly and repeatedly moved up and down between heart level and a height of 35 cm below heart level. Results show that the values of BL in Figure 17a vary in a manner similar to the values of *X*-axis acceleration in Figure 17b and PPG signals tends to be highly affected by the VHD variation.

## 6. Discussion

The values of BL and AM from PPG signals can vary from one person to another due to the finger sensor probe’s size, its placement, effects of body position, individual differences and even differences between the two arms. A PPG signal involves a relatively high frequency component (typically in the range 1–2 Hz) which synchronizes with the heartbeat as well as various lower frequency components relating to ANS (including respiration, e.g., talking, deep breathing events, and physiological events, e.g., body position variation), blood pressure control (local BP variation near sensor probe arising from vertical height difference and thermoregulation.

**Figure 19:**
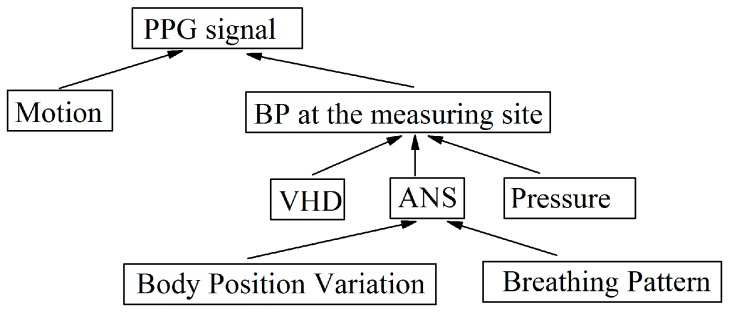
Factors which can affect PPG signals.

A variation in BP corresponding to changing the arm position was measured using the commercial wrist worn BP monitor used in this investigation which was also accompanied by a variation in the PPG signal. A drop of approximately 4 mmHg was measured for each 5 cm rise in VHD, which is in good agreement with the values mentioned in the literature [21,22] which in turn provides efficacy of the results recorded for these parameters in this investigation. However, the purpose of this investigation has been to bring local blood pressure variations into consideration when attempting to remove motion artefact in PPG signals. In summary, factors that may affect the PPG signal during motion have been identified and these are illustrated in Figure 19. External pressure on/near the measuring point, the autonomic nervous system, and the vertical height difference between the measurement site and heart level in the vertical direction, all influence the local BP at the point of measurement. In particular, the induced ANS variations can be as a result of physiological events, including: body position changes (Figure 11), yawn events (Figure 13) and talking events (Figure 14). Also the experimental results indicated that wrist rotation does not significantly affect the PPG signal (Figure 16).

For the PPG signal, it has the properties that the BL of a PPG signal increases with the increase of positive VHD deviation and decreases with the increase of negative VHD deviation. For the case of decreasing of positive and negative VHD deviation, the PPG signal tends to decrease and increase, respectively. The mean and median BLs are derived from the data of Table 2 and Figure 9 and shown in Table 3 and Table 4. The slope of a linear analysis arising from the positive and negative VHD deviation are 0.629 × 10^−3^ cm/V and 0.769 × 10^−3^ cm/V, respectively. Curve fitting for the variation of VHD deviation and BL of PPG would be necessary due to non-linearity but this could be accommodated in the memory of the processing electronics. Therefore, in reverse, the BL of PPG from the finger sensor probe could be used to locate the position of the finger, but this is beyond the scope of this investigation.

The ACC data can be used to infer the finger sensor probe’s direction and acceleration for the analysis of PPG based devices. The PPG finger sensor position can be inferred, from the BL of the PPG signal based on BP variation. PPG-fluctuations can also be used to infer some indication of: (1) a body position change with variation of the finger sensor probe’s position if BL of PPG varies after motion; (2) a body position change without variation of finger sensor probe’s position if BLs of PPG before and after motion are the same; and (3) other physiological variations (e.g., yawn event and deep breathing event). In the context of the existing literature, the results presented in the work of the current investigation are unique and their capture has only been possible due to the high degree of integration of the optical PPG signal detection electronics and accelerometers in one compact unit. Previous work on multi-wavelength PPG signal detection has been performed including some of the authors of this investigation [4], as well as motion artefact reduction (primarily through application of software based post processing [6,7,8]. However, to the best of the knowledge of the authors the in situ accelerometer signals combined with relatively simple algorithms applied to the PPG signals as presented in this article provides a unique opportunity for the possible correction of motion artefact signals in real time.

The pilot study of this investigation PPG level was based on 18 healthy adults and involved 9 males and 9 females. Each participant was required to be subjected to three tests in which the finger sensor probe position relative to the heart was varied (for the sitting position only). Hence, there are a total 54 individual measurements with an effect size of 0.043. The PPG level was measured at the heart level (level 0), 25 cm above heart level (level 1) and 25 cm below heart level (level 2). There is a small but consistent change of PPG amplitude when the vertical height is changed (as is in evidence in Figure 9). In order to study an association between PPG level and vertical height difference (VHD), a multiple linear regression and correlation was performed and the results of this analysis are shown in Table 8. The outcome variable is PPG level and predictor variable are gender and the finger sensor probe positions (level 1 and level 2). The probe position at heart level (level 0) was used as the reference. It can be seen from Table 8 that the p-value for each predictor variable to the outcome is greater than 0.05 (0.361 for gender, 0.521 for level 1 and 0.599 for level 2) which means there is no association between predictor variables to the outcome. In summary, even though there is a discernable change of PPG level according to the vertical height difference (Clear in Figure 9), the height 25 cm above and below the heart level do not significantly affect the PPG level or the blood pressure.

## 7. Conclusions

It has been demonstrated experimentally that the captured PPG signal exhibits sensitivity in its response to the subject’s muscular tension level and limb motion. Devices such as an accelerometer, a respiration monitor belt and a blood pressure monitor, have been deployed in conjunction with the custom designed PPG sensor system for investigation of the effects of external physiological parameters on the PPG signals in the context of autonomic nervous system and local blood pressure. The results have shown that the baseline of PPG signal is affected by acceleration particularly in the vertical direction as opposed to acceleration in the horizontal plane for the system under test. Experimental results from a total of 23 subjects demonstrated that PPG signals during motion events are influenced by the variation of local blood pressure at the point of measurement which is in turn induced from: (1) the vertical height difference between the measuring site and the reference level (the heart level); and (2) the autonomic nervous system. The results of a statistical analysis conducted on 18 subjects (9 male and 9 female) have shown that the dependency of the Baseline (BL) and Amplitude (AM) values of the PPG signal are only weakly dependent on the VHD. It is envisaged that the results of this investigation can be used as a basis for optimizing measurements of PPG based sensors in terms of potential removal of motion artefact.

## Figures and Tables

**Table 1:** Systolic BP, diastolic BP and HR at different height level.

Index	Above Heart Level (cm)	Systolic BP (mmHg)	Diastolic BP (mmHg)	HR (/min)
1	0	112	84	74
2	5	112	79	74
3	10	107	74	74
4	15	103	68	75
5	20	98	65	75
6	25	93	57	75
7	30	91	53	76
8	35	89	45	75

**Table 2:** Mean BLs and AMs at Different VHD Values for 18 individual subjects were in the sitting position and the finger sensor probe set at three different vertical levels.

	Mean Baseline (±SD) (V)			Mean AM (±SD) × 10^−3^ (V)		
Subject	25 cm above Heart Level	Heart Level	25 cm below Heart Level	25 cm above Heart Level	Heart Level	25 cm below Heart Level
1	0.728 (±0.032)	0.705 (±0.027)	0.688 (±0.031)	6.61 (±0.248)	5.21 (±0.213)	5.53 (±0.304)
2	0.378 (±0.024)	0.350 (±0.032)	0.331 (±0.017)	23.64 (±0.823)	13.85 (±0.465)	14.24 (±0.569)
3	0.426 (±0.041)	0.403 (±0.024)	0.378 (±0.029)	22.67 (±0.543)	32.04 (±0.817)	16.87 (±0.481)
4	0.409 (±0.035)	0.352 (±0.030)	0.336 (±0.042)	26.64 (±0.351)	17.01 (±0.721)	16.52 (±0.611)
5	0.543 (±0.022)	0.442 (±0.055)	0.421 (±0.042)	12.30 (±0.267)	22.44 (±0.439)	19.32 (±0.325)
6	0.619 (±0.021)	0.589 (±0.025)	0.550 (±0.026)	6.42 (±0.523)	8.07 (±0.547)	8.44 (±0.413)
7	0.173 (±0.012)	0.165 (±0.011)	0.149 (±0.019)	8.04 (±0.512)	4.44 (±0.391)	5.61 (±0.452)
8	0.426 (±0.049)	0.403 (±0.038)	0.363 (±0.072)	12.57 (±0.745)	13.89 (±0.584)	18.17 (±0.487)
9	0.812 (±0.068)	0.794 (±0.091)	0.765 (±0.048)	8.89 (±0.761)	9.11 (±0.649)	9.66 (±0.946)
10	0.602 (±0.051)	0.684 (±0.077)	0.640 (±0.059)	7.08 (±0.649)	10.57 (±0.381)	10.95 (±0.874)
11	0.962 (±0.021)	0.663 (±0.034)	0.639 (±0.028)	25.25 (±0.915)	23.63 (±0.874)	33.91 (±1.207)
12	0.507 (±0.030)	0.485 (±0.026)	0.448 (±0.047)	9.85 (±0.449)	6.85 (±0.759)	5.09 (±0.618)
13	0.713 (±0.094)	0.652 (±0.028)	0.553 (±0.079)	16.89 (±0.672)	19.58 (±0.364)	15.36 (±0.655)
14	0.478 (±0.035)	0.470 (±0.014)	0.436 (±0.029)	5.75 (±0.493)	9.54 (±0.816)	7.61 (±0.638)
15	0.356 (±0.061)	0.322 (±0.050)	0.277 (±0.043)	17.94 (±0.679)	18.66 (±0.745)	12.08 (±0.483)
16	0.831 (±0.020)	0.812 (±0.048)	0.762 (±0.037)	7.33 (±0.957)	9.04 (±0.461)	6.95 (±0.587)
17	0.647 (±0.051)	0.623 (±0.075)	0.604 (±0.091)	5.39 (±0.518)	6.81 (±0.349)	5.32 (±0.247)
18	0.431 (±0.036)	0.412 (±0.048)	0.401 (±0.067)	10.15 (±0.425)	12.29 (±0.348)	11.58 (±0.591)

**Table 3:** Mean, Median, Standard Deviation, and Interquartile Range of Baseline from 18 sets of PPG signals at Different VHD Deviation.

Statistical Analysis	Baseline of 18 Sets PPG Signal (V)		
	25 cm above Heart Level	Heart Level	25 cm below Heart Level
**Mean**	0.558	0.518	0.487
**Median**	0.525	0.478	0.442
**Standard Deviation**	0.199	0.179	0.173
**Interquartile Range**	0.271	0.257	0.264

**Table 4:** Mean, Median, Standard Deviation, and Interquartile Range of AM from 18 sets of PPG signal at Different VHD Deviation.

Statistical Analysis	AM of 18 Sets PPG Signal × 10^−3^ (V)		
	25 cm above Heart Level	Heart Level	25 cm below Heart Level
**Mean**	12.96722	12.97588	12.40056
**Median**	10	10.57	11.265
**Standard Deviation**	7.293668	7.711909	7.147762
**interquartile range**	10.535	8.94	9.115

**Table 5:** Main parameters of body variation from a standing position to a sitting position.

Subject	*t*_∆ACC_ (s)	*t*_∆BL_ (s)	RR (min^−1^)	∆BL (V)
1	7.23	16.00	20	0.0572
2	5.67	18.12	15	0.0868
3	4.43	17.43	12	0.1107
4	8.44	13.58	18	0.1387
5	5.65	17.16	20	0.1098

**Table 6:** Primary Parameters of Body Variation from Sitting Position to Standing Position.

Subject	*t*_∆ACC_ (s)	*t*_∆BL_ (s)	RR (min^−1^)	∆BL (V)
1	11.36	20.60	18	0.0797
2	9.09	16.23	15	0.0321
3	10.91	16.46	21	0.0499
4	8.63	20.19	18	0.0276
5	11.10	18.52	18	0.0656
6	9.15	19.74	17	0.0434
7	12.06	22.10	23	0.0167
8	10.61	18.84	12	0.0673
9	8.63	19.97	19	0.1287
10	8.49	18.46	19	0.0754
11	10.78	17.26	17	0.0874
12	9.75	18.68	16	0.0756
13	6.88	15.91	22	0.0471
14	10.85	21.32	18	0.0817
15	9.74	19.98	18	0.0459
16	5.26	15.47	16	0.0715
17	8.19	20.62	22	0.0934
18	7.43	16.48	18	0.0692

**Table 7:** Mean, Median, Standard Deviation, and Interquartile Range of Primary Parameters of Body Variation from Sitting to Standing.

Statistical Analysis	*t*_∆ACC_ (s)	*t*_∆BL_ (s)	RR (min^−1^)	∆BL (V)
**Mean**	9.383889	18.71278	18.16667	0.064344
**Median**	9.445	18.76	18	0.06825
**Standard Deviation**	1.762338	2.010536	2.684377	0.026935
**Interquartile Range**	2.3075	3.4625	2	0.032475

**Table 8:** Multiple linear regression and correlation showing the *p*-values of the predictor variables to an outcome (PPG level).

PPG	Coef.	Std. Err.	*t*	*p* > |*t*|	95% Conf.	Interval
gender	−0.0462222	0.050185	−0.92	0.361	−0.1470218	0.0545774
Level 1	0.0397222	0.0614638	0.65	0.521	−0.0837315	0.163176
Level 2	−0.0325	0.0614638	−0.53	0.599	−0.1559538	0.0909538
cons	0.5412222	0.050185	10.78	0.000	0.4404226	0.6420218
